# Ecotoxicity of Fire Retardants to Zebrafish (*Danio rerio*) in Early Life Stages

**DOI:** 10.3390/jox15030079

**Published:** 2025-05-23

**Authors:** Darlan Quinta Brito, Tathyana Benetis Piau, Carlos Henke-Oliveira, Eduardo Cyrino Oliveira-Filho, Cesar Koppe Grisolia

**Affiliations:** 1 Faculty UnB at Planaltina, University of Brasília, Planaltina 73345-010, Brazil; darlanbrito@unb.br; 2 Laboratory of Genetic Toxicology (G-Tox), Department of Genetics and Morphology, University of Brasília, Brasília 70910-900, Brazil; tathyanabenetis@gmail.com (T.B.P.); grisolia@unb.br (C.K.G.); 3 Department of Ecology, Institute of Biological Sciences, University de Brasília, Brasília 70910-900, Brazil; carloshenke@unb.br; 4 Laboratory of Ecotoxicology, Embrapa Cerrados, Planaltina 73310-970, Brazil

**Keywords:** wildfires, fire retardants, *Danio rerio*, embryotoxicity

## Abstract

With the escalating frequency and intensity of global wildfires driven by climate change, fire retardants (FRs) have become essential tools in wildfire management. Despite their widespread use, the environmental safety of newer FR formulations—particularly in relation to aquatic ecosystems and developmental toxicity—remains insufficiently understood. In particular, their effects on fish embryos, which represent a sensitive and ecologically important life stage, are poorly characterized. This study investigated the acute toxicity of three commercially available FRs—N-Borate, N-Phosphate+, and N-Phosphate-—on early life stages of zebrafish (*Danio rerio*), based on an OECD 236 Fish Embryo Toxicity (FET) test. Notably, N-Phosphate- FR exhibited significant toxicity with a 96 h LC50 of 60 mg/L (0.0055%), while N-Borate (>432 mg/L, >0.032%) and N-Phosphate+ (>1181 mg/L, >0.08%) showed substantially lower toxicity. Sublethal effects, including reduced yolk sac absorption and yolk sac darkening, were observed across all FRs, highlighting potential developmental disruptions. The elevated toxicity of N-Phosphate- FR likely stems from its surfactant content. These findings reveal variations in the acute toxicity of different FR formulations, emphasizing the need for ecotoxicological assessments to guide the selection of safer FRs for wildfire management and to protect aquatic biodiversity. The results highlight the importance of incorporating developmental endpoints in FR risk assessments and provide foundational data for regulatory decisions regarding FR application near aquatic habitats. Further research is necessary to elucidate the mechanisms underlying observed effects and to evaluate cross-species toxicity at environmentally relevant concentrations.

## 1. Introduction

A global trend towards warmer and drier environmental conditions, driven by climate change, is amplifying wildfire activity across most regions of the world [1,2,3]. Recent wildfires in the Mediterranean and Middle East [4,5], Australia [6], the United States and Canada [7], and South America [8,9] have garnered attention from governments, scientific communities, and the media due to their devastating impacts on urban infrastructure, public health, biodiversity loss, and natural habitats.

Considering that the frequency and severity of wildfires have increased over the last 40 years, with further escalation projected [10,11], the development and implementation of fire policies and management strategies are imperative. These strategies are crucial for mitigating the adverse effects of wildfires on environmental integrity, human health, and economic stability [12,13]. In pursuit of more effective wildfire suppression techniques, novel fire retardants (FRs), such as foams and gels, have emerged [14,15,16,17].

Fire retardants function by disrupting the combustion triad—fuel, oxygen, and heat—thereby reducing or extinguishing flames, delaying or halting fire progression, and forming a protective barrier to inhibit re-ignition [18,19]. Historically, large-scale applications of FRs containing perfluorinated compounds (PFCs), including perfluorooctane sulfonate (PFOS) and perfluorooctanoic acid (PFOA), have been associated with detrimental environmental effects, characterized by organismal toxicity, bioaccumulation, and environmental persistence [20,21]. The regulatory restrictions on PFC-containing FRs have spurred the development of novel FR formulations which are marketed as environmentally friendly; however, comprehensive knowledge regarding their short- and long-term impacts on environmental systems remains limited [16,22,23].

Although fire retardants (FRs) enhance firefighting effectiveness, their environmental risks must be carefully managed by fire and land agencies [24,25]. In the U.S., the Forest Service prohibits FR application over aquatic systems, protected areas, and critical habitats [26]. Nevertheless, during extreme events like the 2019–2020 megafires in Australia, FRs were applied in sensitive areas, including drinking water catchments and habitats of threatened species [27]. In the absence of national standards concerning the environmental impacts of FRs, Australia recommends using only FRs approved by the USDA [24]. Brazil, like many Latin American countries, has no specific legislation regulating FR use, representing a policy gap that limits the implementation of context-specific regulatory measures [17].

FRs are broadly categorized as long-term or short-term based on their residual effectiveness post-water evaporation. Long-term FRs typically consist of fertilizer salts, such as ammonium phosphate and ammonium sulfate, combined with additives like thickeners, colorants, corrosion inhibitors, and bactericides [28]. Conversely, short-term FRs are designed to enhance the limited fire suppression capacity of water, primarily due to their high surface tension, by incorporating foaming agents, surfactants, foam stabilizers, dispersants, and corrosion inhibitors [29].

While FRs are predominantly applied in terrestrial environments, the unintentional introduction of these compounds into aquatic ecosystems is a serious concern that needs further investigation [26,30]. The aerial application of FRs for wildfire management, particularly in proximity to or over water bodies and small streams, can expose aquatic organisms to elevated concentrations of ammonium (exceeding 100 mg/L) and phosphates, which have been linked to mortality in fish and invertebrates [26,30,31,32]. Furthermore, other proprietary and listed components within FR formulations may contribute to toxicity [16,33,34,35]. To address the knowledge gaps regarding potential FR alternatives, comprehensive ecotoxicological assessments are essential to ensure environmental safety.

Although studies have evaluated the efficacy of FRs [36,37], there is limited information concerning their toxicity to aquatic biota [16,22,23,38]. Moreover, the correlation between observed effects and ecologically relevant parameters, particularly in fish, remains poorly understood. This includes the short-term and sublethal impacts on species most vulnerable to exposure [31,39,40].

Fish are widely used as bioindicators to monitor effluent and surface water quality due to their diverse representation in aquatic ecosystems [41]. *Danio rerio* (Hamilton-Buchanan 1822), commonly known as zebrafish, is a small teleost fish (3.5–4 cm in length) from the Cyprinidae family, inhabiting freshwater environments [42]. Zebrafish are widely utilized as model organisms in toxicological and ecotoxicological research to evaluate the impacts of various chemical compounds on embryogenesis and fish populations [43,44]. This preference is primarily due to their capacity for year-round breeding, high fecundity, rapid developmental processes (approximately 72 h), and the transparency of their chorion, which facilitates toxicity assessments [45,46]. The Fish Embryo Toxicity (FET) test, outlined in OECD Test Guideline 236, has gained increasing global recognition as an effective and sensitive method for assessing the ichthyotoxicity of surface waters [47]. Given the escalating frequency of wildfires and the growing demand for FR usage, understanding the interactions between fish embryos and FR exposure is crucial for the conservation of fish species. Therefore, this study aimed to evaluate and compare the toxicity of three commercially available FRs on the early development of zebrafish. Specifically, we investigated endpoints encompassing lethality, sub-lethality, and teratogenicity to determine whether these compounds induce rapid and severe adverse effects during zebrafish embryogenesis.

## 2. Materials and Methods

### 2.1. Test Chemicals

This study investigated three commercially available fire retardant (FR) formulations selected for their potential application in large-scale wildfire management. Rather than identifying specific brand names, the three products are categorized based on their primary chemical constituents. N-Borate FR is an emerging fire retardant which is formulated as an organo-mineral fertilizer, containing high concentrations of nitrogen and boron, and is classified as long-term [48]; N-Phosphate+ FR is also classified as a long-term retardant, contains elevated levels of nitrogen and phosphorus, and is widely used in aerial wildfire suppression worldwide [26,49]. Its composition is primarily based on ammonium polyphosphate (80–100%), with performance additives (5–10%), attapulgite clay (1–5%), and iron-oxide pigments (1–5%). N-Phosphate− FR is an emerging short-term fire retardant with a similar base composition but with lower concentrations of nitrogen and phosphorus [35,50]. This biodegradable formulation consists of neutralized phosphorus esters, synthetic surfactants, preservatives, and nutrients such as nitrogen, phosphorus, and potassium, being intended for soil and plant applications.

According to the manufacturers’ guidelines for wildfire application, these concentrates are diluted with water to achieve final retardant solutions containing approximately 15% to 20% retardant by volume, with the following specific concentrations for each product: 15% for N-Borate, 15.38% for N-Phosphate+, and 20% for N-Phosphate−. The chemical properties of these formulations are detailed in [35].

### 2.2. Cultivation System and Obtaining of Zebrafish Embryos

Adult zebrafish (5 months) were fed two to three times a day with commercial food (Tetra ColorBits fish food, USA) and live food (nauplii of *Artemia salina)* and were maintained in a facility that used water purified through reverse osmosis and activated carbon filtration (ZebTEC system—Techniplast, Varese, Italy), under a photoperiod cycle of 12:12 h (light: dark) at the Laboratory of Genetic Toxicology of the University of Brasilia (Brazil).

Aquaria were filled with reconstituted deionized water, maintaining conductivity at 740 ± 100 μS/cm, pH levels at approximately 7.0 ± 0.5, hardness at 6.70 dH, temperature at 27 ± 1°C, a photoperiod of 12:12 h light/dark, and dissolved oxygen at 95% saturation. This water system was employed to prepare both the stock solutions and the exposure solutions used in all tests. This study was approved by the Ethics Committee on Animal Use at the University of Brasilia, Brazil (protocol SEI nº 23106.014611/2023-57).

### 2.3. Toxicity Test with Zebrafish Embryos (FET)

The fish embryo test (FET) was based on the OECD protocol No. 236 to assess the lethal concentration (LC_50_) and sublethal effects of the selected FRs on fish embryos [46]. For egg acquisition, male and female fish (2:1 ratio) were placed in the breeding system (i-Spawn), and spawning was triggered once the light came on in the morning. Immediately after spawning, fertilized eggs were collected, rinsed, and examined with a stereomicroscope for viability [45]. Eggs that were unfertilized or exhibited cleavage anomalies or damage were excluded, constituting less than 15% of the total eggs collected.

For the embryo test, 60 eggs per treatment group were utilized and allocated across three 24-well microplates in a climate chamber (SL-24 Solab Científica, Brazil), creating three independent replicates. Twenty wells were filled with the test solution and four wells with system water (internal plate control, as required in the OECD guideline). The stock solution *(v*/*v)* was prepared by diluting FRs in water obtained from the zebrafish facility (physical and chemical characteristics previously described) and was subsequently diluted to obtain the tested concentrations.

Each well was filled with 2 mL of the respective test solution, received one egg, and was prepared at the time of the assay through successive dilutions of the stock solution. The treatments included a control group (using the water from zebrafish system) and six nominal concentration levels of each FR: N-Borate FR (13.5; 27; 54; 108; 216; 432 mg/L; equivalent to 0.001–0.032% *v*/*v*); N-Phosphate+ FR (73.8; 133; 221; 384; 679; 1180.8 mg/L; 0.0025–0.08% *v*/*v*); and N-Phosphate− FR (3.32; 6.65; 13.3; 28; 55; 110.8 mg/L; 0.0003–0.01% *v*/*v*). The final concentrations used in the definitive test were informed by preliminary range-finding assays, which involved exposing embryos to high concentrations of each FR to identify levels associated with mortality. Based on these results, a stepwise dilution scheme (typically a 1:2 ratio) was applied to define six test concentrations per compound.

The microplates were kept in static exposure conditions (27 ± 1 °C and under a 14:10 h light: dark photoperiod cycle) for 96 h.

Zebrafish embryos were monitored throughout the test period at 24, 48, 72, and 96 h post-fertilization (hpf) to evaluate developmental parameters. Prior to hatching, these assessments included egg coagulation, otolith formation, eye and body pigmentation, somite development, heartbeat, the presence of edema, tail bud detachment from the yolk sac, yolk sac absorption, and the hatching process [45,46]. Observations were made using a stereomicroscope (Stemi 2000 C; Carl Zeiss, Oberkochen, Germany).

After hatching, larval observations focused on edema, spinal deformities, and mortality. All parameters were assessed and quantified as observed or not observed. The cumulative lethal concentration (LC50) was determined daily based on the mortality data.

### 2.4. Chemical Analysis

Prior to the static exposure of embryos, water quality parameters were assessed. Water samples from the control, the lowest, and the highest concentrations of each FR treatment were collected, filtered through cellulose ester filters with a pore size of 0.45 μm, and stored at 5 °C for further analysis. The concentrations of water-soluble elements were measured using ion chromatography (Compact IC 761 Metrohm), targeting cations, including lithium (Li^+^), sodium (Na^+^), ammonium (NH_4_^+^), potassium (K^+^), calcium (Ca^2+^), and magnesium (Mg^2+^), and anions, such as fluoride (F^−^), chloride (Cl^−^), nitrate (NO_3_^−^), nitrite (NO_2_^−^), bromide (Br^−^), phosphate (PO_4_^3−^), and sulfate (SO_4_^2−^) [51]. Detection limits for both cations and anions were set at 0.001 mg/L, with the standard ion chromatography range extending from 0.02 to 50 mg/L, using Merck-certified solutions (1000 mg/L). Additionally, the temperature, specific conductance, and pH levels were measured using an AK 88 multiparameter device (AKSO, São Leopoldo, Brazil).

### 2.5. Statistical Analysis

The lethal concentrations (LC50) were determined using a four-parameter logistic model. Differences in hatch rates and sublethal effects (deformities) among the test organisms were assessed through two-way ANOVA to detect intergroup differences for datasets with normal distribution. For datasets that did not meet the criteria for normal distribution, according to the Kolmogorov–Smirnov test, or did not satisfy Levene’s test for homogeneity of variances, the Kruskal–Wallis test was employed instead.

Following the identification of significant differences, Dunnett’s or Dunn’s post hoc test was applied (suitable for both parametric and non-parametric data) to pinpoint significant variations between the control group and each tested concentration (*p ≤* 0.05). All statistical analyses were conducted using SigmaPlot^®^ software, version 14.0 (San Jose, CA, USA).

## 3. Results

### 3.1. Physicochemical Parameters

Water quality parameters, including conductivity and pH, were measured in freshly prepared dilutions at the lowest and highest concentrations of each FR (Table 1). Conductivity and pH levels were similar between the treatments and the control at both the lowest and highest concentrations, except for conductivity in the N-Phosphate+ FR treatment. At the highest concentration, N-Phosphate+ FR exhibited a conductivity value 2.7 times higher than the control and twice that of its lowest concentration. N-Borate and N-Phosphate- FR fully dissolved in the zebrafish medium, while particles from N-Phosphate+ FR tended to precipitate in the beaker. The pH adjustment was made only for the N-Borate FR (Table 1).

In contrast to the total elemental composition of the FRs, as detailed in [35], the analysis of water-extractable ions revealed that phosphate (PO_4_^3−^) was not detected in any of the three FRs. Sulfate (SO_4_^2−^) and ammonium (NH_4_^+^) were the dominant soluble ions in the N-Phosphate+ FR. In the N-Borate FR, sulfate levels were comparable to the control at 0.56 mg/L, while they increased to 4.42 mg/L in the N-Phosphate- FR. However, SO_4_^2−^ levels were substantially higher in the N-Phosphate+ FR, ranging from 18.26 mg/L at the lowest concentration to 90.65 mg/L at the highest.

Nitrogen-based ions exhibited the following trends: Nitrite (NO_2_ˉ) levels were undetectable in all three FRs at both low and high concentrations. Nitrate (NO_3_ˉ) levels remained relatively constant across the three FRs and control. Ammonium (NH_4_^+^) levels increased at the highest concentration, with a 3-fold increase in the N-Phosphate- FR (3.38 mg/L), a 10-fold increase in the N-Borate FR (10.26 mg/L), and a 100-fold increase in the N-Phosphate+ FR (101 mg/L), compared to the control (1.37 mg/L).

### 3.2. Mortality and Hatching Rates of Zebrafish Embryos

**Figure 1:**
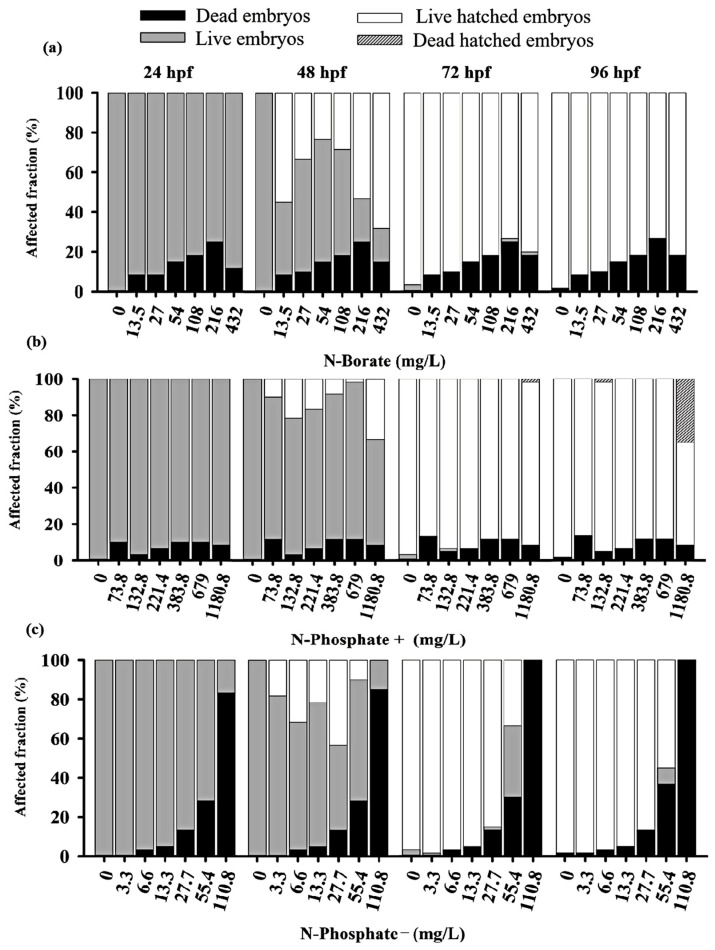
Overview of zebrafish embryos toxicity test at 24, 48, 76, and 96 h for the three fire retardants (FRs) examined: (**a**) N-Borate FR, (**b**) N-Phosphate+ FR, and (**c**) N-Phosphate- FR. The figure displays the proportion of eggs and non-hatched embryos that died as black bars; the proportion of living embryos that remained unhatched as gray bars; those that successfully hatched as white bars; and the proportion of embryos that died post-hatching as spotted and dark dashed gray bars. Data visualization was performed using SigmaPlot^®^ software, version 14.0.

The three FRs exhibited differential impacts on embryo survival before hatching, with the N-Phosphate− FR displaying the highest toxicity compared to the N-Phosphate+ and N-Borate FRs at the tested concentrations (Figure 1).

Adverse effects on zebrafish embryo development were observed throughout the monitoring period. Significant lethal effects, including mortality and failure to hatch by 96 hpf, were recorded. The negative control met OECD guideline No. 236, maintaining a 90% survival rate [46,52,53]. Additionally, pH variations during exposure remained within the tolerance range for zebrafish [46].

A concentration-dependent inhibitory response was observed for the N-Phosphate− FR, with LC50 values of 70.24 mg/L (0.0063%) at 24 h, 69 mg/L at 48 h, 64 mg/L at 72 h, and 60 mg/L (0.0055%) at 96 h (Table S1). The LC50 for the N-Borate FR could not be determined due to the low mortality observed, even at the highest concentration tested, resulting in an estimated LC50 of >432 mg/L (>0.032%) for zebrafish embryos (Table S1). For the N-Phosphate+ FR, the 96 h LC50 exceeded 1180 mg/L (>0.08%) (Figure S1).

Significant mortality was observed for the N-Phosphate− FR across the tested concentrations. After 24 h, the highest concentration (110.8 mg/L; 0.01%) resulted in approximately 82% embryo mortality, reaching 100% by 72 hpf (Figure S2). The elevated mortality associated with the N-Phosphate− FR limited the observation of other potential effects. In contrast, the survival rates of embryos exposed to N-Borate and N-Phosphate+ FRs remained stable, showing no decline throughout the exposure period (Figure 1).

**Figure 2:**
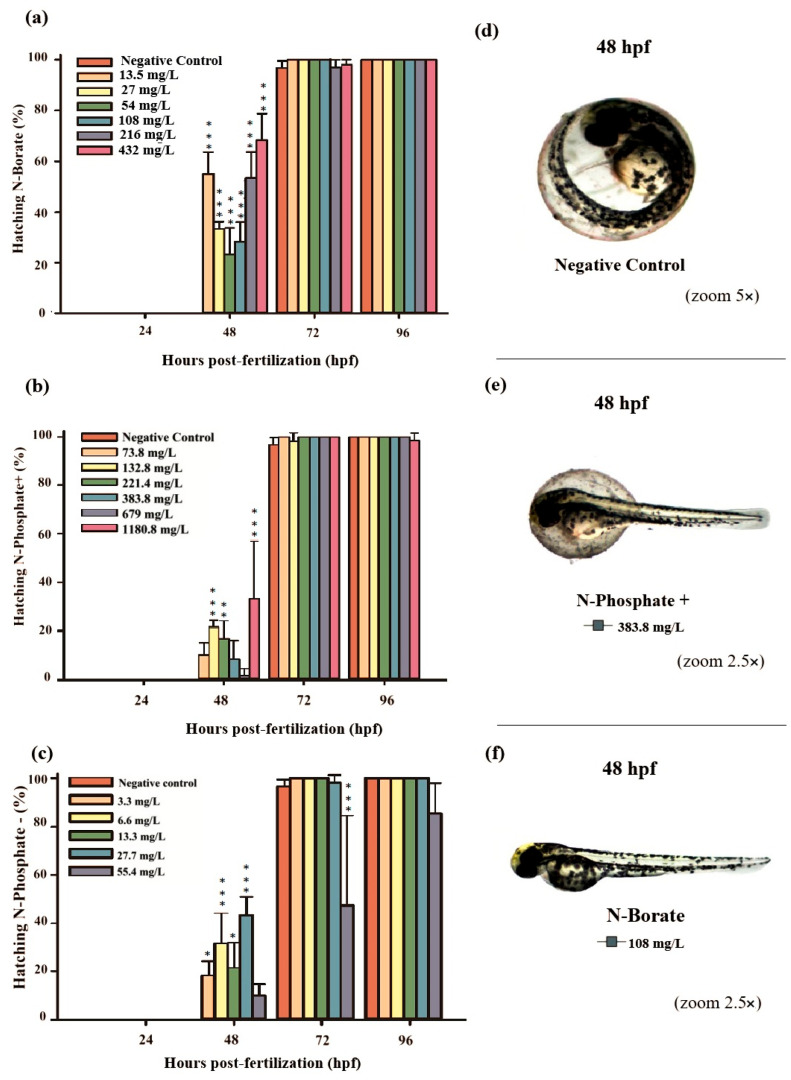
The graphs illustrate the hatching rates during the 96 h exposure period: (**a**) N-Borate FR, (**b**) N-Phosphate+ FR, and (**c**) N-Phosphate- FR. The negative control represents healthy embryos not exposed to fire retardants (FRs). Hatching rates are expressed as mean ± standard error. Statistical significance was determined by comparing exposed groups to the control, with significance levels indicated as follows: * (*p* ≤ 0.05), ** (*p* ≤ 0.01), and *** (*p* ≤ 0.001). Data visualization was performed using SigmaPlot^®^ software, version 14.0. (**d**) Normal embryos, (**e**) partially hatched embryos, and (**f**) hatched embryos at 48 hpf, observed under 5× and 2.5× magnification (Stemi2000-C stereomicroscope).

For the N-Phosphate− FR, a delayed hatching of eggs was observed at 72 hpf, with 20% of the embryos failing to hatch by the end of the observation period. This contrasted with the two other FRs, which did not exhibit such delayed hatching. Notably, most of the embryos that did not hatch by 72 hpf when exposed to N-Phosphate− FR at 55.4 mg/L survived at 96 hpf, suggesting that delayed hatching may not be associated with lethality under these conditions (Figure 1c and Figure 2c).

Premature hatching was significant across all three FRs, affecting approximately 70%, 37%, and 43% of the embryos exposed to N-Borate (432 mg/L), N-Phosphate+ (1180.8 mg/L), and N-Phosphate− (55.4 mg/L), respectively, at 48 hpf (Figure 2a–c).

### 3.3. Teratogenicity of Zebrafish Embryos

**Figure 3:**
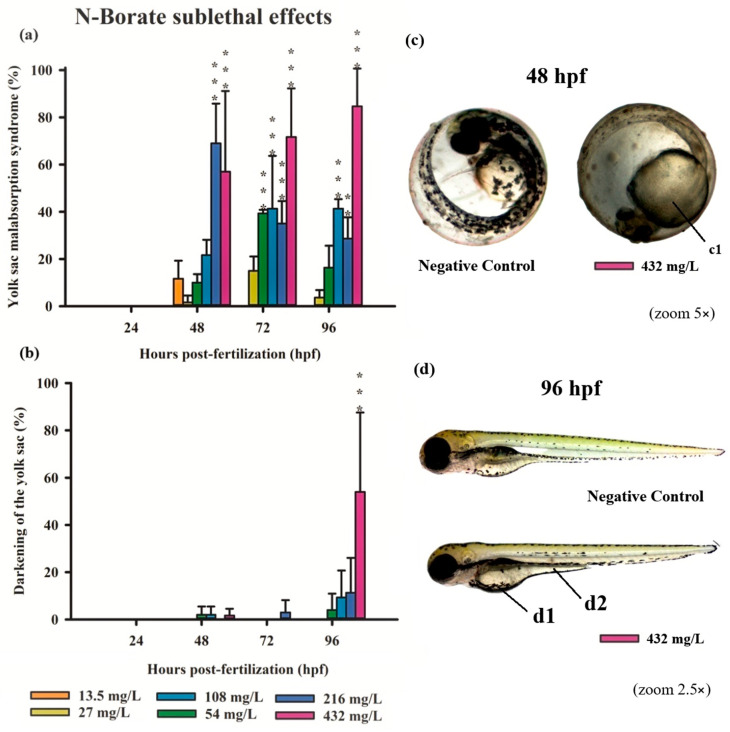
Graphics of sublethal effects induced by different concentrations of N-Borate FR in the zebrafish embryo toxicity test. Specifically, delayed yolk sac absorption (**a**) and yolk sac darkening (**b**) were observed. Statistical analysis was conducted by comparing exposed groups to the control, with significance levels denoted as follows: ** (*p* ≤ 0.01), and *** (*p* ≤ 0.001). Data are presented as mean ± standard deviation. Data visualization was performed using SigmaPlot^®^ software, version 14.0. Sublethal effects observed in embryos exposed to Phosphate- FR (2.5× and 5× magnification, Stemi2000-C stereomicroscope): (**c**) normal embryo and embryo exhibiting delayed yolk sac absorption (c1); (**d**) normal larvae in comparison with larvae showing yolk sac darkening (d1) and delayed yolk sac absorption (d2).

**Figure 4:**
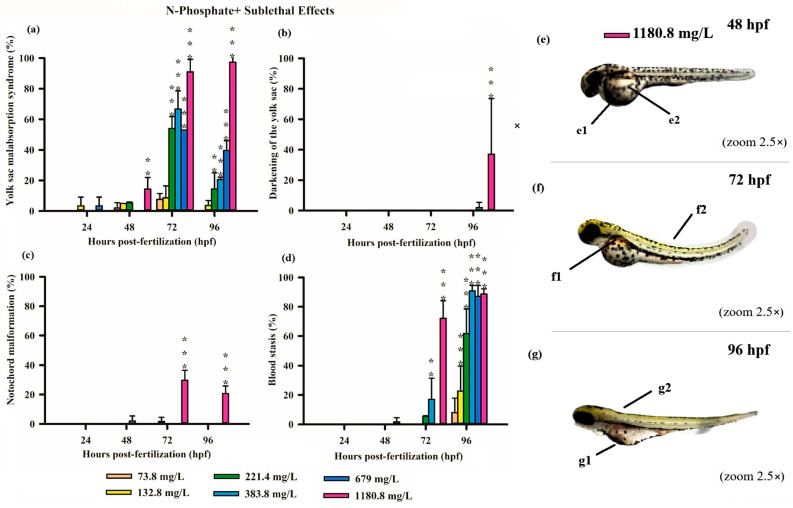
Graphics of sublethal effects induced by different concentrations of N-Phosphate + FR in the zebrafish embryo toxicity assay. Specifically, delayed yolk sac absorption (**a**), darkening of the yolk sac (**b**), notochord malformation (**c**), and blood stasis (**d**) were observed. Statistical analysis was conducted by comparing exposed groups to the control, with significance levels denoted as follows: ** (*p* ≤ 0.01), and *** (*p* ≤ 0.001). Data are presented as mean ± standard deviation. Data visualization was performed using SigmaPlot^®^ software, version 14.0. (**e**–**g**) Sublethal effects observed in embryos exposed to N-Borate FR at a concentration of 1180.8 mg/L up to 96 hpf (2.5× magnification, Stemi2000-C stereomicroscope): e1—yolk sac darkening; e2—delayed yolk sac absorption; f1 and g1—blood stasis; f2 and g2—notochord malformation.

**Figure 5:**
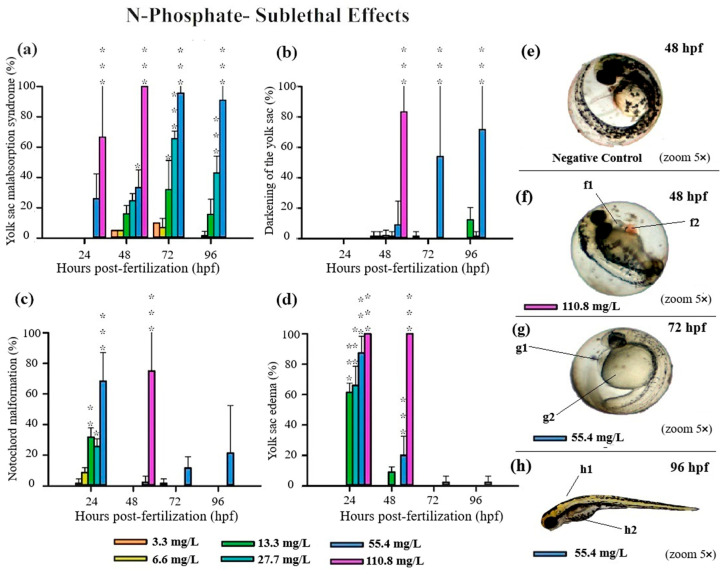
Graphics of sublethal effects induced by different concentrations of N-Phosphate− FR in the zebrafish embryo toxicity test. Specifically, delayed yolk sac absorption (**a**), darkening of the yolk sac (**b**), notochord malformation (**c**), and yolk sac edema (**d**) were observed. Statistical analysis was conducted by comparing exposed groups to the control, with significance levels denoted as follows: * (*p* ≤ 0.05), ** (*p* ≤ 0.01), and *** (*p* ≤ 0.001). Data are presented as mean ± standard deviation. Data visualization was performed using SigmaPlot^®^ software, version 14.0. (**e**–**h**) Sublethal effects observed in embryos exposed to Phosphate- FR (2.5× and 5× magnification, Stemi2000-C stereomicroscope): e—negative control; f1 and g1—yolk sac edema; f2—blood stasis; g2—delayed yolk sac absorption; h1—notochord malformation and h2- darkening of the yolk sac.

All three FRs evaluated in this study induced significant deformities in zebrafish embryos, primarily at higher concentrations (Table S2). Notably, N-Borate FR caused reduced yolk sac absorption and yolk sac darkening (Figure 3). N-Phosphate+ FR led to impaired yolk sac absorption, yolk sac darkening, notochord malformation, and blood stasis (Figure 4). Similarly, N-Phosphate− FR resulted in impaired yolk sac absorption, yolk sac darkening, notochord malformation, and yolk sac edema (Figure 5). Although other morphological deformities were monitored during the experiment, their occurrence was not statistically significant at the tested concentrations.

Exposure to N-Borate FR resulted in a significant reduction in yolk sac absorption in 57%, 70%, and 85% of embryos at 48, 72, and 96 hpf, respectively, at the highest concentration (432 mg/L; 0.032%). In contrast, this effect was observed in 70%, 35%, and 28% of embryos at 48, 72, and 96 hpf, respectively, at a concentration of 216 mg/L (Figure 3a). At a concentration of 54 mg/L, yolk sac absorption impairment was observed in 40% of embryos at 72 hpf, with a significant reduction to 20% at 96 hpf. Furthermore, darkening of the yolk sac was observed only at the highest concentration at 96 hpf (Figure 3b,d).

Similarly, exposure to N-Phosphate+ FR exhibited a concentration-dependent effect on yolk sac absorption, with a trend toward increased yolk sac malabsorption observed at the highest concentration (1180.8 mg/L). In contrast, a reduced degree of yolk sac malabsorption was observed at lower concentrations (221, 384, and 679 mg/L) at both 72 and 96 hpf, although these values remained significantly different from the control (Figure 4a).

Consistent with the findings for N-Borate FR, yolk sac darkening was noted only at the highest concentration at 96 hpf (Figure 4b). Notochord malformation was observed exclusively at the highest concentration at 72 hpf and 96hpf, and blood stasis was detected in 18% of embryos at 679 mg/L and 70% at 1108.8 mg/L at 72 hpf (Figure 4c,d). This effect increased with concentration, reaching 23% at 73.8 mg/L, 60% at 221.4 mg/L, and exceeding 85% at higher concentrations (384, 679, and 1180.8 mg/L) (Figure 4d).

For N-Phosphate-FR, 65% of live embryos exhibited reduced yolk sac absorption at the highest concentration (110.8 mg/L) at 24 hpf, with this effect increasing to 100% of live embryos by 48 hpf (Figure 1 and Figure 5a).

At the highest tested concentration of N-Phosphate- FR (110.8 mg/L), 65% of live embryos (*n* = 7) exhibited reduced yolk sac absorption at 24 hpf, increasing to 100% by 48 hpf (*n* = 11) (Figure 1c and Figure 5a). At 55.4 mg/L, a trend towards an increase in this effect was observed throughout the exposure period, with 30%, 95%, and 90% of embryos being affected at 48, 72, and 96 hpf, respectively (Figure 1c and Figure 5a). Additionally, yolk sac darkening was reported at the highest concentration up to 48 hpf, affecting more than 80% of the embryos (*n* = 8) (Figure 1c and Figure 5b). At 55.4 mg/L, a similar increase in this effect was observed, with 10%, 55%, and 70% of embryos displaying darkened yolk sacs at 48, 72, and 96 hpf, respectively (Figure 1c and Figure 5b).

Notochord malformation was significantly observed at the highest concentration, affecting 75% of the embryos up to 48 hpf. This malformation was also present in embryos exposed to 55.4 mg/L (70%), 27 mg/L (28%), and 13 mg/L (33%) at 24 hpf, although its occurrence notably decreased by 48 hpf and 72 hpf (Figure 1c and Figure 5c).

Yolk sac edema was exclusively observed in embryos exposed to N-Phosphate- FR. This effect was present in 100% of the embryos at the highest concentration at both 24 hpf and 48 hpf. At 24 hpf, yolk sac edema was observed in 87% of embryos at 55.4 mg/L, 65% at 27 mg/L, and 62% at 13.3 mg/L. However, by 48 hpf, this effect diminished significantly, with occurrence rates of 20% at 55.4 mg/L, 0% at 27.7 mg/L, and 10% at 13.3 mg/L (Figure 1c and Figure 5d). No yolk sac edema was observed at the two lowest concentrations (3.3 and 6.6 mg/L) throughout the test.

## 4. Discussion

### 4.1. Acute and Sublethal Toxicity of FRs

Ecotoxicological studies have shown that exposure to certain FRs can lead to significant mortality in aquatic organisms [16,23,31,35]. In the present study, the 96 h LC50—spanning the period of 96 hpf—was estimated to be greater than 432 mg/L (0.032%) for N-Borate FR and 1180 mg/L (0.08%) for N-Phosphate+ FR, suggesting no toxicological risks to the aquatic environment (Table S3). In contrast, N-Phosphate− FR was harmful to zebrafish embryos and larvae throughout the exposure period (Tables S1 and S2). These findings highlight the significant role of the toxicity of FRs in the water following contamination.

Information regarding the aquatic toxicity of N-Phosphate- FR is limited, with available data primarily being derived from the safety data sheet. The reported 96 h LC50 values for this compound include >100 mg/L for adult zebrafish, 48 h EC50 > 100 mg/L for the microcrustacean *Daphnia magna*, and 14-day EC50 >1000 mg/L for the earthworm *Eisenia fetida*. Brito et al. [35] evaluated the same three FRs and identified N-Phosphate- FR as the most toxic to *Ceriodaphnia dubia* (24 h EC50: 56 mg/L; 0.005%; 48 h EC50: 21 mg/L; 0.0019%) and *Daphnia magna* (24 h EC50: 33 mg/L; 0.003%; 48 h EC50: 25.4 mg/L; 0.0023%), highlighting the significant toxicity of newly formulated FRs to daphniid species.

Exposure to sublethal concentrations of all three FRs resulted in various embryonic alterations, including reduced yolk sac absorption, yolk sac darkening, yolk sac edema, blood stasis, and notochord malformation (Figure 3, Figure 4 and Figure 5). Our study demonstrates a correlation between the sublethal effects observed in zebrafish embryos and the presence of these FRs, particularly in the context of co-exposure to metal mixtures. These sublethal effects significantly delayed embryonic growth and development, a critical concern as such delays may impair an organism’s fitness, decreasing its ability to evade predators and forage for food, thus potentially threatening the ecological stability of fish populations [53].

A key challenge in evaluating the variability in acute toxicity is the proprietary nature of FR formulations, as manufacturers often withhold the exact composition of these complex mixtures. Despite claims that N-Borate and N-Phosphate+ FRs are formulated with fertilizer-grade nutrients, concerns regarding their toxicity persist.

### 4.2. Causes of FR Toxicity

The zebrafish is a resilient species, with reported toxic pH thresholds of <5.9 and >8.1 [54]. However, the pH values of the evaluated N-Borate (FR) solutions exceeded the recommended range for zebrafish maintenance (pH 6.5–8.5) [46] prior to adjustment (Table 1). In aquatic environments, borate—a key component of N-Borate FR—dissociates into boric acid and borate anions, with undissociated boric acid being the predominant boron species in freshwater within a pH range of 6–9 [55].

Boron (B) exhibits a U-shaped concentration–response curve in many species [55,56] and is essential for embryonic zebrafish development. Early cleavage-stage embryos are particularly sensitive to B deficiency [54], with adverse effects observed at boron concentrations below 0.497 mg/L [56,57,58].

Acute toxicity studies in fish have shown that the 96 hLC50 for B typically exceeds 200 mg/L. In embryo–larval studies conducted over a period of 4 to 8 days post-hatching, LC50 values for B across six fish species range from 22 to 155 mg/L [55]. Given that the total B content in N-Borate FR is 130.2 g/L (100%) [35], the highest evaluated concentration in this study was approximately 41.66 mg B/L (0.032%) (Figure 1). Therefore, the B concentration in N-Borate FR (41.66 mg/L) is lower than the LC50 value, which can be attributed to either the non-toxicity of this FR at the tested concentration or the potential mitigating effects of co-exposure to the primary metals (B, N, and P).

Currently, ammonium, phosphate and sulfate are considered the primary chemicals of FRs for potential environmental impacts [30,59]. Ammonium-based FRs can introduce significant amounts of (N) and (P) into water bodies, potentially leading to eutrophication [36,59], which may cause oxygen depletion detrimental to aquatic life [60,61,62]. The toxicity of ammonium-based FRs is primarily attributed to ammonia (the sum of NH_3_ and NH_4_^+^), particularly the non-ionized form (NH_3_), which is highly toxic to aquatic organisms [59,62]. Higher pH levels favor the formation of NH_3_, while lower pH promotes the ionized form (NH_4_^+^) [63,64].

In our study, treatments with ammonium-based FRs resulted in elevated N concentrations and increased water conductivity (Table 1). While these factors may not have caused direct toxicity to embryos within the observed concentration range, particularly for N-Borate FR and N-Phosphate+ FR, the potential indirect effects of water quality changes due to FR exposure, such as eutrophication [59] and metal solubility, should be considered in natural scenarios and be further explored in contamination scenarios caused by FRs in aquatic systems.

Studies on the toxic effects of ammonia have identified NH_3_ at concentrations ranging from 0.11 to 22.6 mg/L for freshwater organisms [65]. Reported 24 h LC50 values for NH_3_ range from 0.023 to 0.85 mg/L for various fish species [66,67]. This distinction arises because most biological membranes are permeable to NH_3_ and relatively impermeable to NH_4_^+^ [59].

In rainbow trout (*Oncorhynchus mykiss*), avoidance behavior was observed in response to ammonium-based FRs, including Phos-Chek D75R and Fire-Trol GTS-R, at A concentration of 0.65 mg/L [68]. In contrast, nitrate exhibited lower acute toxicity, with a 96 h LC50 of 1341 mg/L (1010–1607 mg/L) for larval fathead minnows (*Pimephales promelas*) [69]. Another study reported 96 h LC50 values ranging from 228 to 1725 mg/L of NO_3_^−^ for fathead minnow (*P. promelas*), tricolor shiner (*Cyprinella trichroistia*), and tilapia (*Oreochromis* spp.) larvae [70]. In our study, all three FRs evaluated contained NO_2_^−^ and NO_3_^−^ concentrations within the established limits for zebrafish (NO_2_^−^ < 1 mg/L, NO_3_^−^ ≤ 48 mg/L) [53,71]. Growth and developmental delays in fish may result from increased energy demands for ammonia detoxification, reduced food intake due to lethargy, and disruptions in thyroid function or other metabolic processes [63,72]. Nevertheless, the toxic effects of ammonia on the early developmental stages of fish remain incompletely understood [73]. The exposure of fire retardants Phos-Chek LC95W (0.25 and 1 g/L) and BlazeTamer 380 (0.05 and 0.2 g/L) impaired *Limnodynastes peronii* tadpole growth and development over 16 days [22]. Phos-Chek LC95W completely inhibited growth and development, while BlazeTamer380 caused significant delays compared to controls. The greater toxicity of Phos-Chek LC95W was likely due to elevated ammonia levels and associated water quality alterations [22].

The toxicity of FR formulations is commonly attributed to their ammonia content; however, other components, such as colorants, corrosion inhibitors, and proprietary additives, may also contribute to the overall toxic effects. For example, Tunstill et al. [22] reported that the Phos-Chek LC95 A/A-MV formulation, which contains an iron oxide colorant, exhibited higher acute toxicity to *O. mykiss* (96 h LC50 of 370 mg/L) compared to the uncolored version, Phos-Chek LC95W, which had a 96 h LC50 of 470 mg/L.

The toxicity of iron is well documented in the literature, and exposure to ferric ions (Fe^3+^) and ferric oxide nanoparticles (with concentrations ranging from 0.3 mg/L to 10 mg/L) have been shown to cause mortality and cardiotoxicity in zebrafish embryos exposed for 144 h [74]. The cardiotoxic effects of iron, such as those observed with iron oxide nanoparticles or ferric chloride, may have contributed to the formation of blood stasis in N-Phosphate+ FR exposures, as pericardial edema has been previously reported for this element [74].

In our study, the N-Phosphate+ (FR) exhibited a distinct red coloration due to the presence of iron oxide, while N-Borate was blue. In contrast, the N-Phosphate− (FR) was nearly transparent, resembling a detergent-like formulation due to its high surfactant content. These colorants and surfactants may have contributed to the observed toxicological differences among the evaluated FR formulations. Future studies should investigate their potential role in FR toxicity by isolating these compounds to determine their specific effects.

The presence of surfactants in FR formulations may also contribute to toxicity [28]. Previous studies have shown that fire-suppressant foams, such as PC WD-881 and Silv-Ex, exhibit higher toxicity compared to conventional FRs, likely due to anionic surfactants that reduce surface tension and enhance toxic effects [18,74]. Surfactants are recognized as potential toxicants, particularly under anaerobic conditions, as they disrupt cell membrane structure and interfere with survival and development across various aquatic organisms [75,76,77,78,79].

Experimental studies exposing different freshwater fish species to sodium dodecyl sulfate over varying durations (24–96 h) have reported acute ecotoxicity within a concentration range of 8.6–40 mg/L [79]. Although the specific anionic surfactants used in the present study were not identified, the 96 h LC50 for N-Phosphate- FR was 60 mg/L (0.0055%) (Table S1). This concentration is comparable to the 96 h LC50 reported for the egg life stages of rainbow trout (*O. mykiss*) exposed to PC WD-881 and Silv-Ex, which ranged from 44 to over 78 mg/L [18].

The embryo stage is typically less sensitive than the larval stage due to the protective barrier provided by the chorion, which has pores with diameters ranging from 500 to 700 nm. These pores act as a protective barrier for oxygen, nutrient, and waste transport, limiting exposure to external substances [80,81,82,83]. However, the presence of these pores may also facilitate the uptake of toxic compounds, potentially altering the hatching rate and affecting embryo development [84]. In the early hours post-fertilization, the chorion remains permeable. Furthermore, if the molecular size of a compound is sufficiently small, it may penetrate the chorion and exert toxic effects. This appears to be the case with the FR formulations, which were able to traverse the chorion and induce sublethal effects on the embryos (Figure 3, Figure 4 and Figure 5).

Gaikowski et al. [18] assessed the acute toxicity of firefighting chemicals, including fire retardants (Fire-Trol LCG-R, Fire-Trol GTS-R, and Phos-Chek D75-F) and fire-suppressant foams (Phos-Chek WD-881 and Silv-Ex) across five early life stages of rainbow trout (*O. mykiss*). The 96 h LC50 results indicated that eyed eggs were 1.3 to 25.6 times less sensitive to these chemicals than other developmental stages. The swim-up fry, transitioning from endogenous to exogenous feeding, was identified as the most sensitive stage, likely due to its increased susceptibility to stressors introduced by the chemical formulations [18].

At sublethal concentrations, zebrafish embryos and larvae exhibited significant sensitivity to all three FRs, with notable morphological deformities, including reduced yolk sac absorption, yolk sac darkening, yolk sac edema, blood stasis, and notochord malformation (Figure 3, Figure 4 and Figure 5). During early developmental stages, when the organs of the fish are not yet fully functional, external factors such as FR exposure can disrupt normal physiological processes, potentially leading to the formation of edemas [85].

The scoliosis, characterized by an abnormal curvature of the spinal column [52], was observed in larvae exposed to N-Phosphate+ and was more pronounced in those exposed to N-Phosphate− FRs. Additionally, while we reported yolk sac edema induction following N-Phosphate− FR exposure at 24 hpf, our findings indicate edema reabsorption at 48 hpf at the lower concentrations (Figure 5d).

In the present study, a significant increase in premature hatching was observed at 48 h post-fertilization (hpf) in embryos exposed to the highest concentration of N-Borate FR, with 70% of the embryos hatching earlier than those in the control group (Figure 2a). This growth-stimulating effect was most pronounced during the embryonic stage, prior to hatching. Loewengart [55] reported an 8% increase in growth within a boron concentration range of 0.022–0.119 mg/L, with no morphological abnormalities or swimming impairments being observed in embryos or hatched larvae exposed to boron concentrations up to 10.0 mg B/L.

The hatching process in zebrafish is regulated by multiple mechanisms, including embryo movement within the egg, the action of the hatching enzyme chorionase, and osmotic rupture [81]. Prior to hatching, zebrafish embryos develop specialized glands in the head region that produce chorionase, an enzyme that facilitates the disruption of the chorion membrane during the hatching process [86]. The production of chorionase, which is triggered by specific physicochemical signals, also enables the embryo to execute the movements necessary for successful hatching [87,88].

Any delays or inhibition in the hatching process may result from toxic effects that interfere with chorionase activity or prevent the larvae from breaking through the eggshell [89]. Several metals have been shown to influence the hatching process, either by inhibiting or accelerating it in various fish species [90,91,92]. Such delays have been observed in embryos exposed to metals such as Zn, Ni, and Cr for 96 h [93].

### 4.3. Ecological Implications

The environmental effects of FRs can be influenced by site-specific factors, including topography, soil type, cation exchange capacity, stream size, flow dynamics, post-application weather conditions, and water quality parameters like hardness [23,28,36,94,95,96,97]. Despite advances in optimizing FR applications in target areas, accidental inputs of FRs into water bodies can have significant short-term impacts on aquatic ecosystems [28,39,98]. Although documented evidence of ecological disruptions from FR use is limited [26,33], such disruptions may unintentionally occur in small, often temporary water bodies that serve as breeding and developmental sites for various aquatic species [23,39].

The risk of chronic effects from FRs in aquatic environments is generally considered low due to the biodegradation of primary toxic components, such as the conversion of ammonia to nitrate and the breakdown of anionic surfactants into less harmful byproducts [31,39,40,79]. However, our fish embryo toxicity assays indicate that FRs, particularly N-Phosphate-, present acute risks to zebrafish embryos. For example, the field dilution of N-Phosphate- at a ratio of 20 mL per 100 mL *(v*/*v)* yields a concentration of 221,600 mg/L (Table S4).

Comparing field application concentrations of FRs with their respective 96 h LC50 values provides an estimate of the dilution required in receiving waters to reach a concentration lethal to 50% of zebrafish embryos within 96 h [31]. Based on these comparisons, N-Phosphate- FR required a dilution of 3693 times to reach concentrations equivalent to their 96 h LC50 values (Table S3).

The application of FRs in or near small water bodies, whether through deliberate use, runoff, or accidental spills, could have consequences for fish species that breed and develop in these habitats, particularly in the case of N-Phosphate− FR. While the risk of acute toxicity appears to be low for N-Borate and N-Phosphate+ FRs, long-term ecotoxicological data on these formulations are still lacking [22], and they may interact with other environmental contaminants in an additive or synergistic manner.

The observed effects on embryonic development at sublethal concentrations suggest that evaluated FRs may be harmful during critical developmental stages. However, dose-dependent toxicity at higher concentrations emphasizes the need to optimize FR formulations for safe use across varying concentrations. This underscores the importance of conducting dose–response studies to establish safe dosage thresholds, maximizing benefits while minimizing risks.

Variability in toxicity data for FRs hinders direct comparisons across formulations. Further research is needed, including assessments of species from various trophic levels and comprehensive chemical analyses to detect contaminants in FR samples. Despite this, the present study provides valuable insights into the toxicity of FRs used in firefighting, highlighting the need for safer alternatives with similar efficacy. Additionally, these findings emphasize the importance of establishing specific regulations and biomonitoring programs to assess and mitigate the environmental impacts of FRs. Future research should explore the potential of FRs in broader fields, such as environmental remediation. Collaboration between scientists, policymakers, funding agencies, non-academic stakeholders, and industry will be crucial to accelerate the adoption of safer FR formulations, supporting global sustainability efforts [27,99].

Our study assumed simplified water system conditions, treating the water bodies as shallow and stagnant. In natural settings, FRs would likely experience greater dilution in larger or flowing water bodies, potentially reducing the exposure levels for aquatic organisms [28,36]. The lethal concentrations of FRs identified here were determined under controlled laboratory conditions during short-term (96 h) exposures. However, the potential for delayed effects on fish embryos beyond 96 h, as well as impacts on larval behavior, biomarker expression, and responses assessed through omics-based approaches, remains unclear. Furthermore, the influence of environmental variables on FRs warrants further investigation.

## 5. Conclusions

Fire retardants play a critical role in wildfire management; however, the environmental impacts of many formulations remain poorly understood, particularly due to the limited knowledge of their chemical composition. Small lentic water bodies are especially vulnerable to contamination by fire retardants, yet existing ecotoxicological data often overlook sub-lethal effects on aquatic organisms. Our comparative analysis of developmental toxicity indicates that long-term fire retardants (Borate FR and N-Phosphate+ FR) exhibit lower acute toxicity and may represent safer alternatives to N-Phosphate- FR.

Nonetheless, all three formulations were found to disrupt zebrafish embryonic development, including reduced yolk sac absorption and darkening of the yolk sac, suggesting potential long-term consequences for fish growth and survival. These findings highlight the need for further research into the environmental risks associated with fire retardant use, particularly focusing on molecular and other suborganismal responses in embryo–larval zebrafish to better understand the mechanisms driving these effects.

Alterations in pH and the presence of surfactants in fire-retardant formulations may contribute to their aquatic toxicity. Given the limited data on the environmental concentrations of fire retardants, further research is necessary to assess their toxicity at ecologically relevant levels, with particular emphasis on short-term exposure and community-level impacts to guide management practices. Fostering interdisciplinary collaborations between academia, industry, and policymakers will be essential to fully realize the potential of fire-retardant technologies, ensuring their role in contributing to a more sustainable future in firefighting efforts.

## Figures and Tables

**Table 1:** Solubilization of the cations and anions (mg/L) of negative control and the lowest and highest concentration for the three fire retardants (FRs) used in the fish embryo test (FET).

Fire Retardant Type		N-Borate		N-Phosphate+		N-Phosphate−	
Concentration mg/L (%)	Control	Lowest 13.5 (0.001)	Highest 432 (0.032)	Lowest 74 (0.005)	Highest 1181 (0.080)	Lowest 3.32 (0.0003)	Highest 111 (0.01)
F^−^	<DL	<DL	<DL	<DL	<DL	<DL	<DL
Cl^−^	65.54	69.96	81.04	59.69	68.54	62.45	64.17
NO_2_^−^	<DL	<DL	<DL	<DL	<DL	<DL	<DL
Br^−^	0.25	0.26	0.32	0.17	0.30	0.24	0.30
NO_3_^−^	9.48	10.14	10.2	7.37	9.64	9.55	9.61
PO_4_^3−^	<DL	<DL	<DL	<DL	<DL	<DL	<DL
SO_4_^2−^	0.56	0.57	0.57	18.26	90.65	0.88	4.42
Li	<DL	<DL	<DL	<DL	<DL	<DL	<DL
Na^+^	69.74	74.78	74.9	55.28	70	68.67	65
NH_4_^+^	1.37	0.38	10.26	23.15	101	<DL	3.38
K^+^	2.97	3.22	3.05	2.46	3.73	3.02	2.95
Ca^2+^	5.58	6.05	6.45	4.83	5.94	5.93	5.80
Mg^2+^	8.10	8.80	9.0	6.41	8.71	7.80	7.64
pH	7.1	6.98	7.2 *	6.97	7	7.25	7.55
Conductivity (μS.cm^−1^)	442	442	562	583	1196	443	483

<DL stands for below detection limit (<0.001 mg/L). * Before pH adjustment the pH for N-Borate was 9.2.

## Data Availability

The original contributions presented in this study are included in the article. Further inquiries can be directed to the corresponding author.

## Supplementary Materials

The following supporting information can be downloaded at https://www.mdpi.com/article/10.3390/jox15030079/s1, Table S1. LC_50_ (mortality) values for zebrafish embryos exposed to three fire retardants (FRs) at different exposure times; Table S2. Teratogenic effects observed in zebrafish embryos exposed to three fire retardants (FRs); Table S3. Based on LC50 values obtained by the assays, the compounds were classified as established by the Globally Harmonized System of Classification and Labeling of Chemicals (GHS); Table S4. Concentrations of the three fire retardants (FRs) in the field-relevant mixture and the corresponding ratios of mixture concentration to the zebrafish embryo (LC_50_) for each exposure day. Figure S1. Concentration-response curve (mortality) of organisms exposed to N-Phosphate+ FR for 96 h revealing an LC50 of 2723.7 mg/L—Model: sigmoidal—4 parameters. R^2^ = 0.87. Figure S2. Mortality parameters observed in zebrafish embryos and larvae following a 96-h exposure to N-Phosphate- FR. Graphic shows the mortality rates, expressed as the proportion of coagulated eggs and the absence of heartbeats, comparing exposed organisms to the negative control group of healthy embryos unexposed to N-Phosphate- FR.
