# Supplementary material for: Ecotoxicity of Fire Retardants to Zebrafish (Danio rerio) in Early Life Stages
Source: J Xenobiot. 2025 May 23;15(3):79. doi: 10.3390/jox15030079 (PMC12194123; doi:10.3390/jox15030079)
Supplement: Supplementary file 1 [file jox-15-00079-s001.zip › jox-3576041-supplementary.pdf]

Ecotoxicity of Fire Retardants on Zebrafish (*Danio rerio*) Survival and Development

Darlan Quinta de Brito[1*]; Tathyana Benetis Piau[2]; Carlos Henke-Oliveira[3]; Eduardo Cyrino Oliveira-Filho[4*]; Cesar Koppe Grisolia[2]

[1] Faculty UnB at Planaltina, University of Brasília, Planaltina, Federal District (DF), Brazil. darlanbrito@unb.br (D. Q. B.)

[2] Department of Genetics and Morphology, Institute of Biological Sciences, University of Brasília, Brasília, Brazil. tathyanabenetis@gmail.com (T.B.P); grisolia@unb.br (C.K.P)

[3] Department of Ecology, Institute of Biological Sciences, University de Brasília, Brasília, Brazil. carloshenke@unb.br (C. H. O.)

[4] Embrapa Cerrados, Laboratory of Ecotoxicology, Planaltina, Federal District (DF), Brazil.

*Corresponding author: eduardo.cyrino@embrapa.br (E.C.O.F.)

Table S1. LC_{50} (mortality) values for zebrafish embryos exposed to three fire retardants (FRs) at different exposure times.

Fire retardant type	24h-LC50 (%) (mg/L) *	48h-LC50 (%) (mg/L)	72h-LC50 (%) (mg/L)	96h-LC50 (%) (mg/L)
**N-Borate**	ND	ND	ND	ND
**N-Phosphate+**	ND	ND	ND	ND
**N-Phosphate-**	0.0063 (0.0054 - 0.0073) 70.24 (60 - 81)	0.0063 (0.0054 - 0.0073) 69 (60 - 81)	0.0058 (0.0051- 0.0068) 64 (56 - 75)	0.0055 (0.0047- 0.0064) 60 (52 - 71)

*LC_{50} values are presented as % exposure with 95% confidence intervals in parentheses, followed by equivalent concentrations in mg/L [CI]. ND = Not determined due to mortality not exceeding 50% in any treatment. LC_{50} values calculated based on manufacturer-reported densities.

Table S2. Teratogenic effects observed in zebrafish embryos exposed to three fire retardants (FRs).

Fire retardant type	Hatching Effects	Observed Malformations	Concentration (%) (mg/L)
**N-Borate**	PH	lys, dys	0 - 0.032<br>0 - 432
**N-Phosphate+**	PH	lys, dys, not, bs	0 - 0.08<br>0 - 1,180
**N-Phosphate-**	PH (48 h), DH (72 h)	lys, dys, not, yse	0 - 0.01<br>0 - 110.8

DH - delayed hatching. **PH -** premature hatching; M- Malformations: **lys -** low yolk sac absorption; **dys** - darkening of yolk sac; **yse -** yolk sac edema, **not -** notochord malformation, and **bs -** blood stasis.

Table S3. Based on LC50 values obtained by the assays, the compounds were classified as established by the Globally Harmonized System of Classification and Labeling of Chemicals (GHS).

Degree of toxicity of FRs	LC50 (mg/L)
Highly toxic	≤ 1
Toxic	1 ≤ 10
Harmful to aquatic organisms	10 ≤ 100
Nontoxic	> 100

Table S4. Concentrations of the three fire retardants (FRs) in the field-relevant mixture and the corresponding ratios of mixture concentration to the zebrafish embryo (LC_{50}) for each exposure day.

Fire retardant	Field mixture		Ratio of field mix to:			
Type	Standard(%)[a]	mg/L[b]	24-h LC50	48-h LC50	72-h LC50	96-h LC50[c]
**N-Borate**	15	202,500	NA	NA	NA	>469
**N-Phosphate+**	15.4	227,304	NA	NA	NA	>192
**N-Phosphate-**	20	221,600	3,154	3,211	3,462	3,693

[a] Weight or volume of chemical concentrate (%) combined with water to produce a solution recommended by the manufacturers.

[b] Calculated based on the density provided by the manufacturers, as shown in table 1.

[c] Ratio: field mixture/median lethal concentration (LC50) (mg/L).

NA: Not available due to embryo´s mortality not exceeding 50% in any treatments.

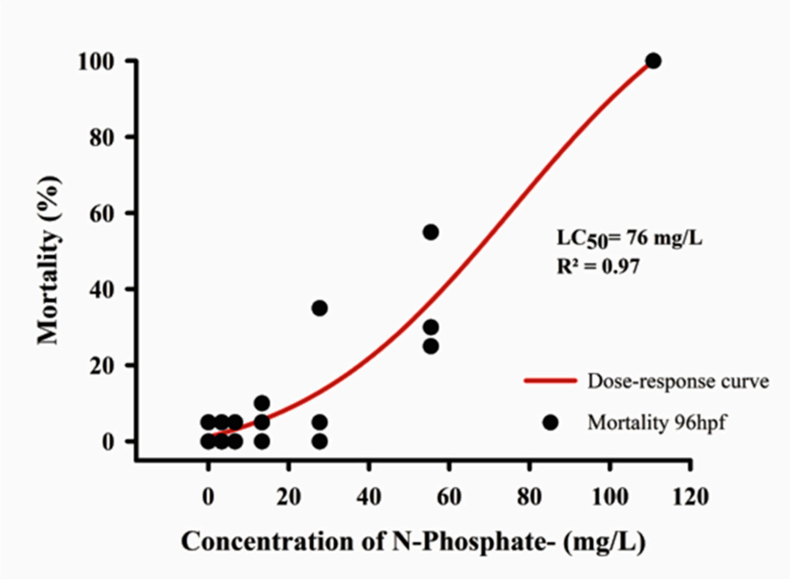

Figure S1. Concentration-response curve (mortality) of organisms exposed to N-Phosphate+ FR for 96 h revealing an LC50 of 2723.7 mg/L—Model: sigmoidal—4 parameters. $R^2 = 0.87$.

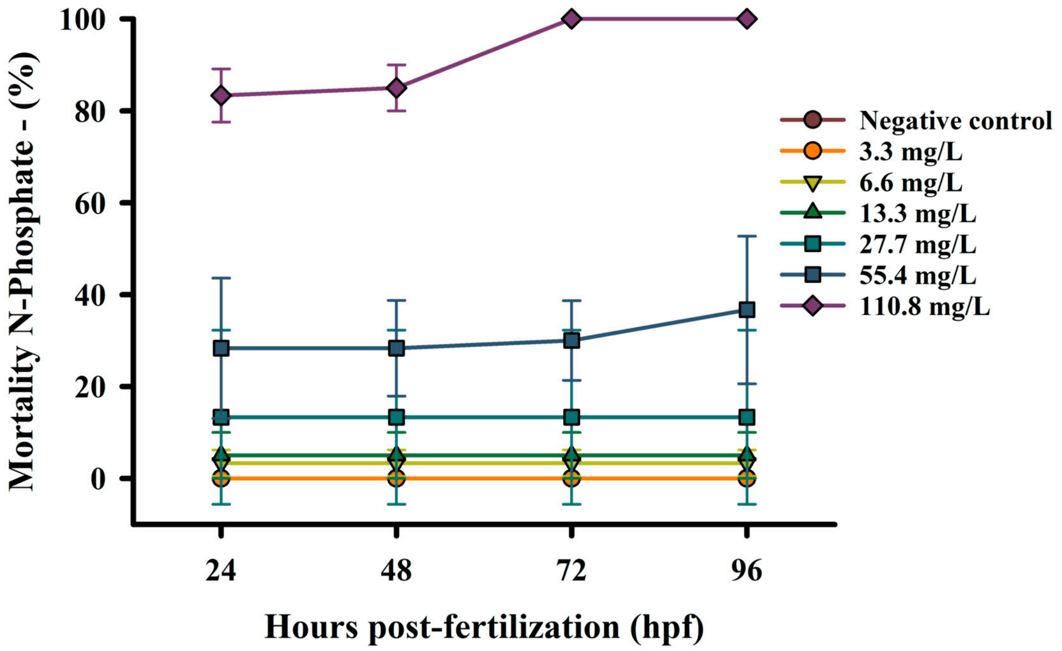

Figure S2. Mortality parameters observed in zebrafish embryos and larvae following a 96-h exposure to N-Phosphate- FR. Graphic shows the mortality rates, expressed as the proportion of coagulated eggs and the absence of heartbeats, comparing exposed organisms to the negative control group of healthy embryos unexposed to N-Phosphate- FR.
